# Electroacupuncture for poststroke urinary incontinence: A systematic scoping review

**DOI:** 10.1097/MD.0000000000045884

**Published:** 2026-01-09

**Authors:** Tao Jiang, Shiyi Jiang, Ying Cui, Ji-Peng Yang, Yuan-Hao Du, Jing Li, Bo Pang, Bo Li, Yuping Ma, Junpeng He

**Affiliations:** a National Clinical Research Center for Chinese Medicine Acupuncture and Moxibustion, Tianjin, China; b Department of Acupuncture and Moxibustion, First Teaching Hospital of Tianjin University of Traditional Chinese Medicine, Tianjin, China; c Department of Rehabilitation, Xishan District Traditional Chinese Medicine Hospital, Wuxi, China; d Tianjin University of Traditional Chinese Medicine, Tianjin, China; e Evidence-based Medicine Center, Tianjin University of Traditional Chinese Medicine, Tianjin, China.

**Keywords:** electroacupuncture, poststroke urinary incontinence, scoping review

## Abstract

**Background:**

Electroacupuncture has gained considerable prominence in treating poststroke urinary incontinence. However, there is no systematic review on current topics and clinical findings on electroacupuncture for poststroke urinary incontinence. This scoping review was conducted to systematically summarize and overview the prevalent themes and research evidence of electroacupuncture for poststroke urinary incontinence.

**Methods:**

PubMed, the Cochrane Library, Web of Science, Embase, CNKI, VIP, Wan Fang Data, and SinoMed databases were searched from inception to June 1, 2023. Relevant literature on electroacupuncture for poststroke urinary incontinence were identified. Two researchers filtered studies and extracted the information independently. Quantitative analysis and descriptive approach were used. The scoping review was conducted according to the PRISMA-ScR checklist.

**Results:**

A total of 58 publications were included, comprising 49 clinical studies, 2 reviews, 5 systematic reviews, and 2 conference abstracts. Chinese researchers conducted all the clinical studies and published them from 2008 to 2022. Electroacupuncture had definite therapeutic effects in treating poststroke urinary incontinence from various outcome measures. In the treatment of acupuncture for poststroke urinary incontinence, multiple commonly employed or recommended acupuncture points were identified in the context of electroacupuncture, including EX-HN1, BL23, RN3, RN4, BL32, SP6, GV20, RN6, and BL35. Notably, all the studies demonstrated promising outcomes, characterized by a statistically significant reduction in the severity of urinary incontinence and the commendable efficacy of electroacupuncture intervention.

**Conclusion:**

Electroacupuncture as a secure and efficacious alternative intervention showed substantial therapeutic potential in treating poststroke urinary incontinence. Further exploration necessitates additional large-scale, well-designed studies and international collaboration to facilitate the comprehensive clinical evaluation of electroacupuncture therapy in poststroke urinary incontinence.

## 1. Introduction

Urinary incontinence, defined as involuntary loss of urine,^[1]^ represents a prevalent manifestation of poststroke voiding dysfunction,^[2]^ with an incidence ranging from 28 to 79% among poststroke patients.^[3]^ The principal etiology of this disorder stems from uncontrolled bladder contractions and detrusor muscle hyperactivity, both stemming from neurological pathways impairments.^[4]^ Prolonged and recurrent urinary incontinence can precipitate detrimental outcomes such as pressure ulcers and urinary tract infections.^[5]^ Moreover, the occurrence of poststroke urinary incontinence imposes not only a psychological burden on patients but also exerts significant financial strain on their families.^[6]^ Thus, early identification and efficacious management of poststroke urinary incontinence are essential.

Clinically, the common management of poststroke urinary incontinence includes pharmacological interventions and behavioral strategies.^[7,8]^ First, Anticholinergic agents have gained recognition and widespread utilization as the pharmacological approach.^[9]^ Their primary mechanism of action involves inhibiting detrusor muscle overactivity and inducing smooth muscle relaxation in the bladder. However, the clinical use of prescription drugs is limited due to their significant adverse effects, including xerostomia and constipation.^[10]^ Focusing on bladder function and pelvic floor muscle training has a special relieving effect on ameliorating poststroke urinary incontinence. Nonetheless, effective implementation of rehabilitation training necessitates patient compliance and adherence to prescribed regimens.^[11]^

Acupuncture is a commonly employed therapeutic modality for poststroke symptoms, including urinary incontinence.^[12]^ Due to the low level of stimulation, traditional acupuncture is unable to directly induce proprioceptive impulses in the bladder and is difficult to achieve the required treatment threshold, and the therapeutic benefit is modest.^[12,13]^ Electroacupuncture, an amalgamation of ancient Chinese medical acupuncture and contemporary treatment approaches, offers the advantages of cost-effectiveness and user-friendly application, rendering it a viable option for managing poststroke urinary incontinence. Studies^[14,15]^ have demonstrated that electroacupuncture elicits cortical regulation of the urinary center, inhibits detrusor muscle overactivity and neurotransmitter release, promotes blood circulation, facilitates meridian clearance in the bladder, enhances pelvic floor muscle function, coordinates urinary activity, and promotes bladder contractility and relaxation.^[16]^

Scoping review serves as an initial step in literature research, employing the principles of evidence-based practice to systematically identify and organize research evidence. Its purpose is to offer a comprehensive understanding of existing studies’ quantity, attributes, and characteristics across diverse research areas while also providing potential applications.^[17]^ The primary objective of a scoping review is to provide a descriptive overview of the available literature, incorporating a more thorough and systematic search to present a broad and varied synthesis of research about general topics. In the context of investigating electroacupuncture for poststroke urinary incontinence, conducting a scoping review may be a suitable approach to explore the existing evidence comprehensively.^[18]^

The primary aim of this scoping review was to elucidate clinical investigations and reports concerning the utilization of electroacupuncture as a treatment modality, thereby furnishing clinicians, patients, and researchers with in-depth insights into the application of electroacupuncture for poststroke urinary incontinence. Furthermore, this review sought to foster the advancement of clinical research in this field by offering comprehensive information and promoting further exploration.

## 2. Methods

The Preferred Reporting Items for Systematic Reviews and Meta-Analyses (PRISMA)^[19]^ and PRISMA Extension for Scoping Reviews (PRISMA-ScR) guidelines (Supplementary File 1, Supplemental Digital Content, https://links.lww.com/MD/Q861)^[20]^ were followed for this scoping review. The following 5 stages were used to conduct this review in accordance with Arksey and O’Malley methodological approach for scoping reviews^[18,20,21]^: identification of the research questions, identification of the relevant articles, selection of studies for review, data extraction and charting, and summary of the results.

### 2.1. Review questions

The following questions were addressed during this scoping review:

1. What are the most common acupoints used to treat poststroke urinary incontinence?
2. Who is suitable for this type of electroacupuncture treatment?
3. What are the main theoretical and therapeutic characteristics of electroacupuncture for poststroke urinary incontinence?
4. What are the results of these electroacupuncture treatments for urinary incontinence?

### 2.2. Eligibility criteria

Relevant studies encompassing randomized controlled trials (RCTs), controlled clinical trials (CCTs), case series, reviews, and systematic reviews were systematically identified, irrespective of language, publication form, or publication status. Inclusion criteria for poststroke clinical trials necessitated the inclusion of patients diagnosed with poststroke urinary incontinence based on standardized diagnostic criteria^[7]^ while excluding those with cognitive impairment, mental disorders, and significant underlying comorbidities. Additionally, studies involving patients with severe systemic or neurological diseases, urinary tract infections, undergoing preoperative radiotherapy or chemotherapy, or presenting a combination of significant risks such as cardiovascular, liver, kidney, and hematopoietic disorders, as well as those refusing acupuncture treatment, were excluded due to the potential confounding effects of these factors. Interventional trials employed electroacupuncture as a standalone treatment or combined with other active interventions. Noninvasive techniques like laser acupuncture or transcutaneous electrical stimulation were not considered in the interventions. Exclusion criteria encompassed literature unrelated to electroacupuncture treatment, personal opinions, magazines, news bulletins, duplicate publications, and sources that could not be accessed.

### 2.3. Literature search

A systematic search was conducted across 8 English and Chinese electronic databases, namely PubMed, Web of Science, Cochrane Library, Embase, China National Knowledge Infrastructure (CNKI), VIP, Wan Fang Data, and SinoMed, covering the period from their inception to June 1, 2023. To ensure a comprehensive search, specific search strings were developed for each database, incorporating terms such as electroacupuncture, electroacupuncture therapy, stroke, poststroke, urinary incontinence, and involuntary urination. These search terms were applied to titles, abstracts, and keywords of relevant articles (Supplementary File 2, Supplemental Digital Content, https://links.lww.com/MD/Q861). The reference lists of identified papers were also screened to identify additional pertinent studies.

### 2.4. Study selection and data extraction

Two review authors (TJ and SYJ) independently conducted separate searches across English and Chinese electronic databases. To eliminate duplicate studies, SYJ utilized Endnote X9 software. Following the screening of titles and abstracts, reports meeting the eligibility criteria were selected for further inclusion. TJ and SYJ critically assessed the potentially eligible articles for final selection. In the event of any disagreements, they were resolved through discussion or by consulting the third review author (BP).

The 2 authors (TJ and SYJ) independently extracted relevant information into a predetermined form and conducted a thorough cross-check to ensure accuracy. In cases of any discrepancies, they engaged in discussion and reached a consensus. The form, following the Preferred Reporting Items for Systematic Reviews and Meta-Analyses (PRISMA) guidelines and the PICO (population, intervention, comparison, outcome) framework, included details such as the first author, publication year, study type, sample size, gender, age, interventions, outcomes, and other pertinent data.^[22]^ A descriptive analysis was conducted using the collected information, computing frequencies, and percentages. Microsoft Excel software was utilized to create graphical representations of the data.

## 3. Results

**Figure 1.**
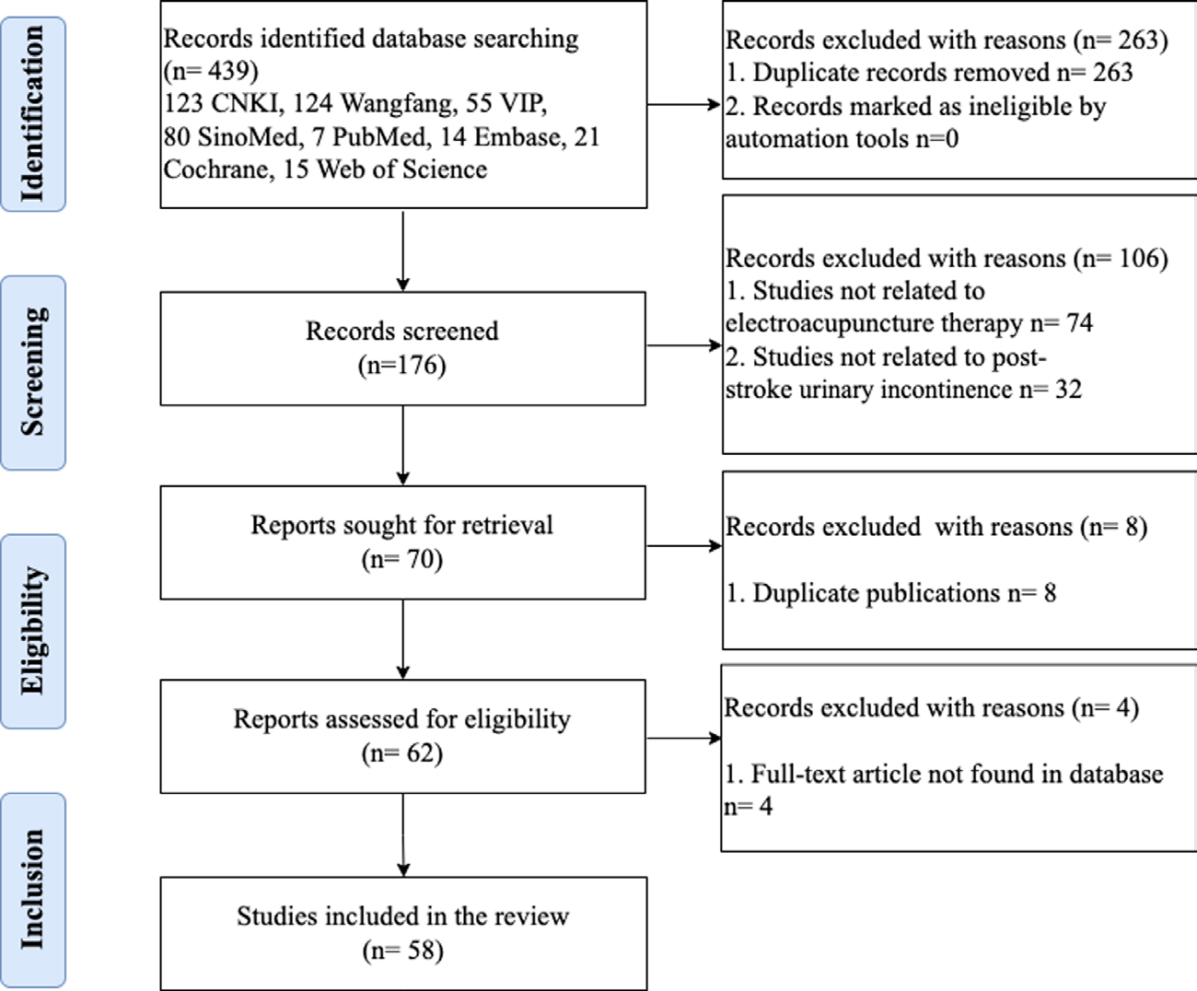
Process and results of literature screening.

The preliminary investigation yielded a total of 439 pertinent reports, with 382 published in Chinese and 57 in English. After removing duplicate literature, 176 research articles remained for further evaluation. Through the screening of titles and abstracts, 106 records were excluded as they did not align with the study topic, resulting in 70 studies eligible for further assessment. Duplicate publications and articles without accessible original sources were also excluded. Ultimately, 58 eligible articles were retained for analysis. Figure 1 outlines the search and selection process. Out of the 58 included studies, 46 (79.3%) were RCTs,^[14–16,23–65]^ while 3 (5.2%) were case series,^[66–68]^ 2 (3.4%) were reviews,^[12,13]^ 5 (6.8%) were systematic reviews, and 2 (3.4%) were conference abstracts. The number of published articles exhibited a consistent annual increase. The earliest RCT investigating electroacupuncture for poststroke urinary incontinence was published in 2008. Since 2011, RCTs have become the predominant study design. All 49 clinical studies were conducted in China from 18 different provinces. These studies were published in 33 distinct journals, with 47 (95.9%) in Chinese and 2 (4.1%) in English, with the Journal of Clinical Acupuncture and Moxibustion (7, 14.3%) being the most prevalent.

### 3.1. Overview of clinical research

A total of 4129 patients were enrolled in the 49 clinical studies, with sample sizes ranging from 30 to 218 (median: 78, mean: 84.3). All patients included in poststroke clinical trials, both in the experimental and control groups, were diagnosed with poststroke urinary incontinence based on standardized diagnostic criteria. Patients with cognitive impairment, mental disorders, and severe underlying comorbidities were excluded from these studies. Among the included studies, 43 (87.8%) provided information on the age of participants, which ranged from 40 to 86 years. A summary of the clinical studies is presented in Table 1.

**Table 1:** Characteristics of included clinical research.

Study	Types of research	N (*T*/*C*)	Age (*T*/*C*)	Intervention	Control	Acupuncture points	Courses	Outcomes
Feng 2011	RCT	30/29	60–78	Electroacupuncture, routine treatment and bladder function training	Routine treatment and bladder function training	Motor sensory areas of the foot, BL23, BL28, BL32, BL30, BL35	4 wk	*,†
Chen 2020	RCT	55/55	68.97 6.44/69.53 ± 7.21	Electroacupuncture	Manual acupuncture	Four points of caudal sacrum	4wk	*,‡,§
Yu 2011	RCT	28/28	62.4 ± 4.63/63.7 ± 3.46	Electroacupuncture	Manual acupuncture	Motor sensory areas of the foot, RN6, RN4, RN3, BL31, BL32, BL33, BL34	2 wk	*,‡,∥
Chen 2011	RCT	33/30	66.21 ± 10.86/64.87 ± 8.29	Electroacupuncture	Chinese medicine treatment	BL31, BL32, BL33, BL34	4 wk	*,†,¶
Wang 2022	RCT	80/80	40–75	Electroacupuncture and routine treatment	Routine treatment	RN4, RN3, KI2	2 wk	‡,∥,#
Chen 2015	RCT	60/60	60.83 ± 7.54/66.10 ± 7.80	Electroacupuncture	Routine treatment	EX-HN1, BL23, BL35	2 wk	*,†,#
Chen 2008	RCT	16/16	66.27/67.81	Electroacupuncture	Drug treatment and bladder function training	BL32, BL33, BL35	4 wk	*
Liu 2010	RCT	30/30	67.62/68.62	Electroacupuncture	Drug treatment and bladder function training	BL32, BL33, BL35	4 wk	∥
Song 2013	RCT	136/68	55 ± 7/54 ± 7	Electroacupuncture	Bladder functional treatment and indwelling catheter treatment	CV2, RN3, ST28, RN6, GV20, EX-HN1, ST36, SP6	4 wk	†,∥,#
Song 2009	RCT	60/60	60.53/60.18	Electroacupuncture	Drug treatment	CV2, RN3, ST28, RN6, GV20, EX-HN1, BL32, BL23	4 wk	*
Song 2011	Case series	218	/	Electroacupuncture	/	CV2, RN3, ST28, RN6, GV20, EX-HN1, BL32, BL23	8 wk	*
Liu 2007	RCT	102/51	46–86/52–82	Electroacupuncture	Drug treatment	BL32, BL33, BL35	4 wk	*
Wang 2017	RCT	35/35	/	Electroacupuncture	Drug treatment	GV20, EX-HN1, BL23, BL35	20 d	*,#
Liu 2020	RCT	20/20	54.9 ± 8.44/57.1 ± 12.17	Electroacupuncture and routine treatment	Routine treatment and drug treatment	RN3, RN4	4 wk	†,‡,∥,#
Li 2014	RCT	31/31	61.5	Electroacupuncture	Routine treatment	EX-HN1, BL23, BL35	4 wk	*,#
Zhao 2013	RCT	15/15	54.47 ± 6.35/55.67 ± 7.66	Electroacupuncture	Manual acupuncture	Motor sensory areas of the foot, BL31, BL32, BL33, BL34	2 wk	*,∥
Zhang 2009	Case series	60	/	Electroacupuncture	/	Motor sensory areas of the foot, BL32, BL35	2 wk	∥,#
Zhao 2007	RCT	54/54	>45	Electroacupuncture	Manual acupuncture	BL28, RN3, EX-HN1, BL62, SP6, SP9, CV5, RN5	30 d	*,#
Yang 2006	RCT	30/30	50–80	Electroacupuncture	Chinese medicine treatment	EX-HN1, BL23, BL35	2 wk	*,#
Chu 2011	RCT	56/55	66.36 ± 9.32/64.5 ± 8.63	Electroacupuncture	Routine treatment	EX-HN1, BL23, BL35	4 wk	*,¶,#
Su 2011	RCT	30/30	52/55	Electroacupuncture and routine treatment	Chinese medicine treatment and routine treatment	EX-HN1, BL23, BL35	2 wk	†,∥
Wang 2004	Case series	128	/	Electroacupuncture	/	RN3, CV5, RN5, EX-HN1, LR3, KI3, BL62, SP6, SP9	40 d	*
Li 2017	RCT	22/20	60.0 ± 9.2/58.5 ± 8.7	Electroacupuncture	Electroacupuncture	BL32, BL23	2 wk	†,∥
Sun 2020	RCT	30/30/30	61.3 ± 7.82/62.03 ± 7.59/61.73 ± 8.48	Electroacupuncture	Manual acupuncture	Motor sensory areas of the foot, corresponding projection area of bladder	2 wk	*,¶,#
Zhou 2012	RCT	46/46	59.8 ± 15.2/60.1 ± 14.7	Electroacupuncture and routine treatment	Routine treatment and drug treatment	GV20, EX-HN1, RN4, RN3, ST28, BL23, BL28, BL32, SP9, SP6, KI3	4 wk	*,†,#
Li 2019	RCT	30/30	54.23 ± 2.32/53.46 ± 2.63	Electroacupuncture, routine treatment and moxibustion	Electroacupuncture and routine treatment	RN4, RN6, RN3, ST28	2 wk	*,¶,#
Wang 2011	RCT	43/43	45–66	Electroacupuncture and pelvic floor muscle exercise	Pelvic floor muscle exercise	GV20, RN3, RN4, ST36, SP6, KI3	3 mo	*
Zhao 2016	RCT	72/72	61.21 ± 5.44/60.45 ± 5.06	Electroacupuncture and pelvic floor muscle exercise	Pelvic floor muscle exercise	GV20, RN3, RN4, ST36, SP6, KI3	3 mo	*,†,¶,#
Lin 2020	RCT	30/30	57.53 ± 9.53/55.93 ± 8.06	Electroacupuncture and manual acupuncture	Manual acupuncture	CV2, RN3, ST28, RN6	4 wk	†
Jiang 2020	RCT	30/30	61.5 ± 7.3/62.5 ± 8.0	Electroacupuncture, moxibustion and Chinese medicine	Chinese medicine treatment	Motor sensory areas of the foot, BL23, BL35	2 wk	*,†,‡,¶,#
Fang 2016	RCT	45/45	64.53 ± 7.78/65.16 ± 8.13	Electroacupuncture, routine treatment and moxibustion	Indwelling catheter treatment	GV20, EX-HN1, RN4, RN3, SP9, SP6	2 wk	*,‡,¶
Zeng 2022	RCT	48/48	60.70 ± 4.40/58.40 ± 5.20	Electroacupuncture, routine treatment and elongated needle therapy	Electroacupuncture and routine treatment	RN3, BL28, BL23, SP6, ST28	2 wk	*,†,∥,#
Zeng 2022	RCT	52/52	43 ± 6/43 ± 6	Electroacupuncture and routine treatment and elongated needle therapy	Electroacupuncture and routine treatment	BL32, SP6, RN3	28 d	*,†,‡,∥
Xu 2009	RCT	40/38	50–80	Electroacupuncture and drug treatment	Routine treatment	EX-HN1, BL23, GV1	2 wk	*,#
Zhang 2012	RCT	50/47	43–75	Electroacupuncture and pelvic floor muscle exercise	Pelvic floor muscle exercise	GV20, RN3, RN4, RN6, SP8, SP6, BL23, BL28	2 wk	*,†
Miao 2014	RCT	30/30	54.83 ± 6.45/54.83 ± 6.45	Electroacupuncture, warming acupuncture and moxibustion	Electro stimulation and pelvic floor muscle exercise	BL31, BL32, BL33, BL34, BL23, SP9	3 wk	*,#
Jiang 2012	RCT	29/29	50–80	Electroacupuncture and acupoint application	Biofeedback therapy instrument treatment	RN4, RN3, SP6, GV4, BL23, BL28	6 wk	†
Xu 2019	RCT	32/32	65.55 ± 2.74/65.59 ± 2.37	Electroacupuncture, warming acupuncture and moxibustion	Chinese medicine treatment	GV20, EX-HN1	2 wk	*,**
Yu 2019	RCT	40/40	63.35 ± 9.78/60.63 ± 10.89	Electroacupuncture, warming acupuncture and moxibustion	Routine treatment	EX-HN1, RN4, RN6	8 wk	§,#
Di 2020	RCT	30/30	58.61 ± 2.74/58.45 ± 2.63	Electroacupuncture, warming acupuncture and moxibustion	Routine treatment	EX-HN1, RN4, RN6	40 d	§,∥,#
Song 2008	RCT	40/40	60.83 ± 9.95/59.8 ± 9.58	Electroacupuncture	Drug treatment	CV2, RN3, ST28, RN6, GV20, EX-HN1, BL32, BL23	2 wk	*,**
Xing 2019	RCT	41/41	41.25 ± 4.53/40.98 ± 4.46	Electroacupuncture	Pelvic floor muscle exercise	BL23, BL35	2 wk	†,¶
Xin 2014	RCT	20/20	/	Electroacupuncture and acupoint injection	Manual acupuncture	GV20, RN3, RN4, BL22, BL28, SP6, BL32	30 d	*,#
Dong 2020	RCT	30/30	46.8 ± 17.2/45.6 ± 15.6	Electroacupuncture, pelvic floor muscle exercise, bladder function training and rtms	Pelvic floor muscle exercise, bladder function training and rtms	GV20, EX-HN1, RN4, RN3, SP9, SP6	4 wk	†,‡
Hu 2011	RCT	43/43	55–86/56–84	Electroacupuncture, moxibustion and Chinese medicine	Electroacupuncture	BL31, BL32, BL33, BL34	8 wk	*,†
Qiao 2019	RCT	44/44	62.13 ± 3.47	Electroacupuncture, warming acupuncture and moxibustion	Routine treatment	EX-HN1, RN4, RN6	2 mo	§,#
Zhou 2017	RCT	15/15	52–70	Electroacupuncture and moxibustion	Routine treatment	GV20, EX-HN1, RN4, RN3, SP9, SP6	2 wk	*,‡,§
Sun 2010	RCT	34/34	60.38 ± 4.35/59.12 ± 4.62	Electroacupuncture, routine treatment and bladder function training	Routine treatment and bladder function training	RN8, RN4, RN6, RN3, SP6, LR8	40 d	*,†,‡,∥
Lu 2019	RCT	40/40	/	Electroacupuncture, routine treatment, warming acupuncture and moxibustion	Routine treatment and manual acupuncture	EX-HN1, RN4, RN6	8 wk	#

* Therapeutic effects.

† Urodynamic determination.

‡ Number of urine leakage.

§ ICIQ_UISF scores.

∥ Number of urinations.

¶ Clinical symptom scores.

# Degree of urinary incontinence.

** Patients’ satisfaction.

22 (44.9%) studies^[14–16,23–28,30,34,35,41,43,44,47,53,56,59,66,68,69]^ employed electroacupuncture as a most common intervention, whereas the remaining studies incorporated combinations of electroacupuncture with routine treatment,^[33,38,55,62]^ functional training^[36,40,46,54]^ or other active treatment modalities. The control groups predominantly received drug treatment, manual acupuncture, or a combination of drug treatment and functional exercises. The most commonly employed control form was drug treatment, including traditional Chinese medicine interventions.

**Figure 2.**
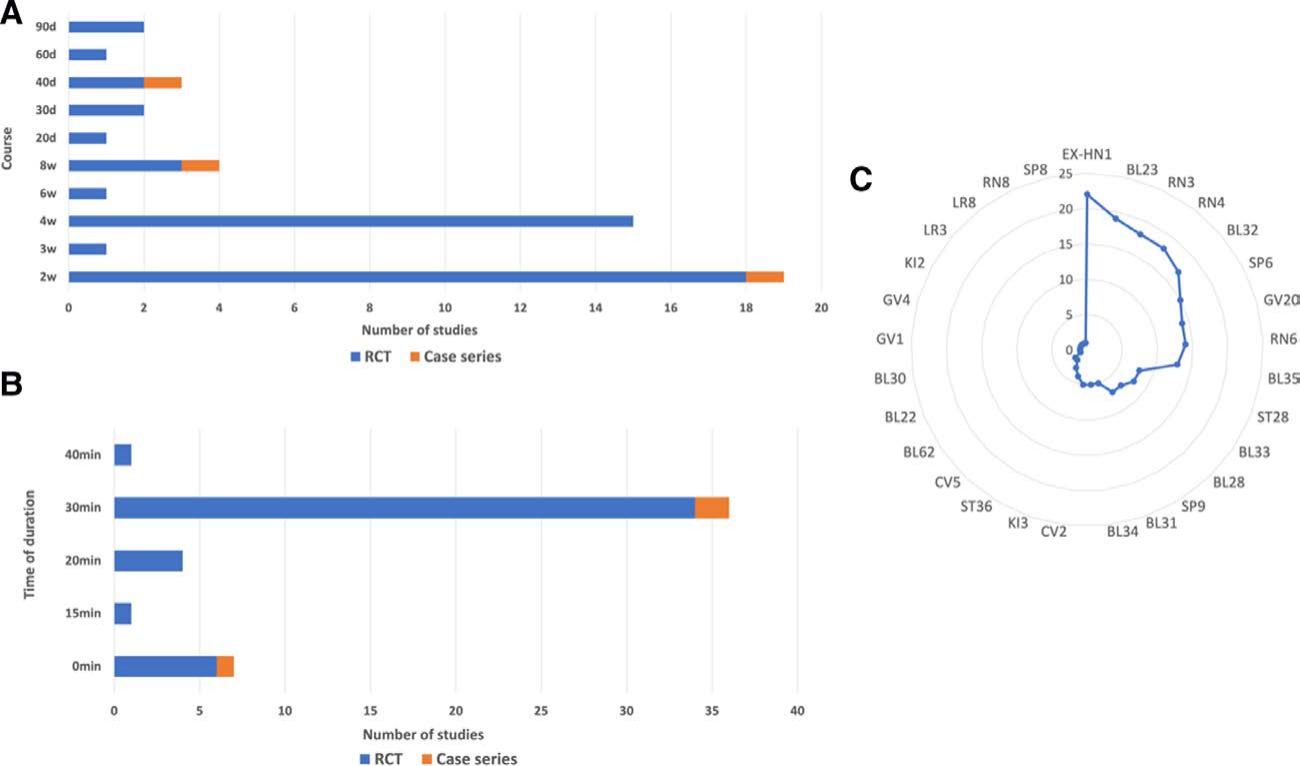
Characteristics of treatment and acupoint selection of electroacupuncture for poststroke urinary incontinence. (A) Treatment courses. d = day(s), w = week(s). (B) Time of duration of each session (statistics of clinical research); min = minutes; (C) frequency of selection acupoints in clinical research.

Among the included studies, 8 studies provided specific details regarding the choice of needle type, including specifications such as length, diameter, and brand/material. Stainless steel acupuncture needles measuring 0.3*40mm were the most commonly utilized. Furthermore, 21 studies described the precise depth at which the acupuncture needles were inserted into the designated acupoints. The duration of treatment sessions varied across studies, ranging from 2 weeks to 3 months. Regarding the time of needle retention, 46 studies reported durations ranging from 20 minutes to 40 minutes, with a majority (36 studies, 73.5%) employing a duration of 30 minutes (Fig. 2). Electroacupuncture served as the primary intervention in the majority of studies, with 33 (67.3%) studies of them providing details on the model or brand name of the electroacupuncture device. Only 7 (14.3%) studies did not specify the frequency or waveform of electroacupuncture utilization, among other factors. Within the included studies, 17 (34.7%) studies used sparse or dense waves.

The primary outcome measures encompassed various indicators, including therapeutic effects, degree of urinary incontinence, number of urine leakages, International Consultation on Incontinence Questionnaire Short Form (ICIQ_UISF) scores, urodynamic assessments, number of urinations, clinical symptom scores, and patients’ satisfaction. Therapeutic effects (37, 75.5%) emerged as the most frequently employed outcome measure across the studies to evaluate the impact of electroacupuncture on urinary incontinence treatment. Additionally, the comparison of the degree of urinary incontinence (22, 44.9%) served as another effective outcome measure in the studies. The clinical studies indicated that electroacupuncture therapy exhibited precise efficacy in treating poststroke urinary incontinence, showcasing a clinical effectiveness rate surpassing 80% and noteworthy amelioration in the degree of urinary incontinence. Merely 5 studies employed ICIQ_UISF scores as an outcome measure. ICIQ_UISF scores, being a globally recognized urinary incontinence assessment scale, accurately portray the disparity between pre-and posttreatment conditions in clinical research, thereby underscoring the significant capacity of electroacupuncture in ameliorating poststroke urinary incontinence.

### 3.2. Characteristics of treatment and acupoint selection

In general, the findings from clinical studies have demonstrated the favorable efficacy of electroacupuncture in managing poststroke urinary incontinence. Significant improvements have been observed in various parameters, including the number of urine leaks, clinical symptoms, and scores indicating the degree of incontinence. Among the analyzed publications, a total of 12 (24.5%) studies reported on the safety profile of electroacupuncture treatment. Specifically, 6 studies provided information on side effects associated with electroacupuncture. Within these studies, 3 reported subcutaneous bleeding or hematoma formation, which resolved spontaneously. Additionally, 2 studies highlighted pain as the primary adverse reaction. Notably, 1 study documented the unfortunate occurrence of a patient’s death; however, it was determined that the cause of death was unrelated to acupuncture.

Regarding the selection of acupuncture points, the frequently utilized or recommended points in clinical trials were EX-HN1 (22, 44.90%), BL23 (19, 38.78%), RN3 (18, 36.73%), RN4 (18, 36.73%), BL32 (17, 34.69%), SP6 (15, 30.61%), GV20 (14, 28.57%), RN6 (14, 28.57%), and BL35 (13, 26.53%). SP6 can tonify qi and blood in the 3 meridians, promote the relaxation of tendons and channels, strengthen the spleen and stomach, and tonify the liver and kidneys. It aids in enhancing the bladder’s restraining function and replenishing the qi of the spleen and kidneys. GV20 serves as the converging point of meridians, connecting peripheral meridians and influencing all yin and yang channels. It regulates yin and yang, awakening the brain, opening the orifices, benefiting qi, fixing detachment, and restoring the bladder’s restraining function. RN4 can warm yang, fix detachment, and tonify the kidneys. It contributes to the adjustment of the bladder’s restraining function, with additional effects of clearing dampness and heat, tonifying kidney qi, stimulating the bladder, and addressing the root cause of the condition.^[46]^ RN6 functions as the convergence site of the body’s vital energy, regulating water metabolism, tonifying the kidneys, enhancing qi, regulating the 3 jiaos, and promoting urinary cessation.^[57]^ Electroacupuncture at BL32 can effectively open meridians, promote blood circulation, eliminate blood stasis, facilitate the flow of qi and blood, nourish yin and yang, optimize the function of the guanqiao, and restore urinary function.^[37]^

## 4. Discussion

This study conducted a systematic scoping review to assess the current state of electroacupuncture in poststroke urinary incontinence. It identified supportive evidence for the use of electroacupuncture in the treatment of poststroke urinary incontinence and provided supplementary reliable, and transparent evidence for upcoming clinical research.

### 4.1. Advantages of electroacupuncture for poststroke urinary incontinence

First, electroacupuncture represents an alternative therapeutic modality, distinct from pharmacological interventions, due to its diminished incidence of adverse reactions and enhanced overall safety profile. Electroacupuncture is more commonly utilized than medicine for poststroke urinary incontinence, as acupuncture treatment bypasses hepatic and renal metabolism, making it easily applicable, cost-effective, safe, and well-tolerated.^[70]^ Consequently, it emerges as an appealing treatment method, particularly for patients with contraindications or intolerance to specific medications.

Acupuncture exerts a biphasic effect on bladder capacity, bladder pressure, and the regulation of bladder compliance. Acupuncture points’ neuronal pathways overlap with bladder afferent neurons, influencing the regulation of the sacral medullary micturition center through the peripheral bladder and urethral afferent or efferent nerve activity. Consequently, acupuncture not only inhibits bladder function but also stimulates smooth muscle activity. This dynamic balance between inhibition and stimulation helps maintain appropriate urine storage and voiding function in the bladder.^[71–73]^ Animal experiments and clinical studies have demonstrated that electroacupuncture treatment effectively inhibits excessive detrusor muscle contractions, induces detrusor relaxation, increases bladder volume and voiding pressure, and improves bladder compliance, thereby restoring bladder function.^[74]^

In contrast to traditional acupuncture, electroacupuncture synergistically integrates the advantages of acupuncture and electrical stimulation, offering sustained and potent stimulation to enhance therapeutic efficacy over prolonged durations.^[75]^ Studies^[14,41]^ demonstrated that uninterrupted electroacupuncture stimulation exerted a progressive influence on the external and internal urethral sphincter muscles, engendering continual augmentation of muscle strength and tone. Consequently, this amplified the inhibitory effect on micturition by intensifying urethral sphincter contraction. Furthermore, the incessant electrical stimulation of electroacupuncture fosters localized hemodynamic circulation, ameliorates neural trophism, facilitated the regenerative potential and repair of partially functional or suppressed nerve fibers, and expedited the conduction of nerve impulses.^[41]^ Thus, it engendered neural functional restoration and promoted the resolution of urinary disturbances, constituting an efficacious approach for managing poststroke urinary incontinence. A meta-analysis^[76]^ demonstrated that electroacupuncture significantly alleviates urinary incontinence symptoms following stroke, with patients achieving better clinical outcomes compared to control groups. Electroacupuncture also improved associated complications, enhanced bladder sphincter control, and reduced the incidence of urinary tract infections, ultimately improving patients’ quality of life.

### 4.2. Consensus on etiological and therapeutic mechanisms

In addition to clinical studies, the 12 literature included various subjects, reviews, systematic reviews, protocols, and conference abstracts. They comprehensively explored the underlying causes and intricate mechanisms associated with poststroke urinary incontinence in the context of electroacupuncture treatment.

According to the principles of traditional Chinese medicine, a consensus has been sought regarding the etiological and therapeutic mechanisms of poststroke urinary incontinence. The pathological site of poststroke urinary incontinence lies within the bladder.^[13]^ In the early stage of a stroke, under the fierce attack of pathogenic factors, the body’s vital energy rapidly declines, leading to even collapse. In the later stage, due to the failure of vital energy to recover, the invasion of phlegm-dampness pathogenic factors affects the Bladder’s function of Qi transformation, resulting in uncontrolled urination and potential urinary incontinence. The occurrence of this condition is closely related to the Kidney and is also closely associated with the lungs, spleen, and liver. The pathological nature of the condition is rooted in deficiency but manifests with excess symptoms. Specifically, Lung-Spleen insufficiency, Liver-Kidney deficiency, and instability of the Lower Jiao are the underlying causes, while the accumulation of phlegm-dampness and stagnant blood in the Bladder are the presenting features. Therefore, in traditional Chinese medicine treatment for poststroke urinary incontinence, emphasis is placed on addressing both the root cause and the presenting symptoms. It involves replenishing Qi and supporting the body’s vitality while eliminating bladder phlegm and stasis, focusing on localized treatment.^[12]^

Poststroke urinary incontinence is widely recognized in modern medicine as a sophisticated and multifactorial condition encompassing a myriad of intricate mechanisms. These encompass dysregulation of the detrusor muscle, compromised functionality of the urethral sphincter, diminished sensory perception within the bladder, and neurological impairments that extend to the central and peripheral nervous systems. The genesis of poststroke urinary incontinence can be attributed to the disruption of the higher center responsible for the cerebral voiding reflex, consequently resulting in a diminished inhibitory impact on the primary center of the voiding reflex. As a result, uncontrolled bladder contractions ensue, culminating in the manifestation of urinary incontinence.^[12]^ Moreover, the emergence of poststroke urinary incontinence is primarily correlated with the precise site of cerebral injury, with lesions affecting regions intricately involved in regulating bladder control, such as the frontal cortex, basal ganglia, and brainstem, being identified as pivotal factors. Additionally, the onset and recovery of poststroke urinary incontinence are not exclusively contingent upon the anatomical location and size of the lesion but are significantly influenced by the severity of the underlying condition, age-related factors, and a multitude of other determinants. These cumulative factors exacerbate bladder dysfunction and substantively contribute to initiating or persisting urinary incontinence after a stroke.

### 4.3. Consideration of treatment method for poststroke urinary incontinence

Patients who meet the diagnostic criteria for poststroke urinary incontinence, excluding cognitive impairment, urinary tract infection, or significant hepatic or renal disorders, and who are suitable for electroacupuncture treatment can undergo electroacupuncture therapy to ameliorate poststroke urinary incontinence. Electroacupuncture integrates traditional acupuncture with modern physiotherapy techniques, providing the most effective treatment modality.^[12]^ According to research findings, the predominant approach in electroacupuncture involves needling specific acupoints, achieving the sensation of “De Qi,” and applying electroacupuncture stimulation for a duration not exceeding 30 minutes. Typically, acupuncture sessions are administered once daily, 5 times per week, with a treatment duration ranging from 2 weeks to 3 months. Low-frequency continuous waves ranging from 1 to 100 Hz are selected for electroacupuncture, generating uninterrupted current to stimulate the internal and external urethral sphincters. This stimulation enhances muscle strength and tone, leading to increased inhibition of urination through urethral contraction. Studies have demonstrated the effectiveness of low-frequency micro-currents in modulating bidirectional regulation at acupuncture points, particularly in intervening with the detrusor muscles of the bladder.^[77]^ Furthermore, combining electroacupuncture with bladder function training has shown enhanced efficacy. Clinical studies have reported a success rate exceeding 90 percent with the combination of electroacupuncture and bladder function training for poststroke urinary incontinence.^[31,53]^ Sjöström M et al.^[78]^ revealed that functional exercise targeting the pelvic floor muscles facilitates the transmission of proprioceptive motor and sensory information to the brain’s central nervous system. This promoted the recovery of corresponding central nervous functions, ultimately enhancing control over the pelvic floor muscles for treating or managing urinary incontinence.

Regarding acupoint selection, recommended choices include EX-HN1, BL23, RN3, RN4, BL32, and SP6. The paracentral lobule beneath GV20 and EX-HN1 corresponds to the cortical motor center for voiding, where electroacupuncture stimulation effectively activates the cerebral cortex and restores inhibition of the sacral medullary voiding center. RN3, RN4, RN6, and BL35 encompass the subcutaneous distribution of the inferior ventral nerve, pelvic nerve, and pubic nerve. Acupuncture at these points regulates the relaxation and contraction of the external urethral sphincter, reducing urinary frequency. BL23 not only stimulates sympathetic nerves and inhibits bladder detrusor contractions but also promotes internal urethral sphincter contractions, thereby increasing bladder capacity and reducing urination frequency. BL32 corresponds to the location of the sacral nerve, and acupuncture at this point directly stimulates the sacral medulla, improving bladder function.^[38]^

### 4.4. Implications for research

Published RCTs have indicated the historical development and ongoing advancements in the use of electroacupuncture for poststroke urinary incontinence. While case series have provided some real-world evidence of effectiveness, they cannot serve as conclusive evidence.^[79]^ However, these RCTs often lacked rigorous scientific design, appropriate statistical analysis, and uniform criteria for assessing efficacy. Additionally, few studies employed internationally recognized therapies as control groups or utilized internationally recognized urinary incontinence scales for evaluating efficacy,^[80]^ which limits our ability to determine the exact effectiveness and superiority of electroacupuncture. Furthermore, there is a lack of long-term follow-up studies on the efficacy and safety of electroacupuncture for poststroke urinary incontinence, preventing the verification of its long-term effectiveness.^[81]^

The selected studies demonstrated variations in acupoint selection, needle manipulation techniques, duration of needle retention, and treatment duration for electroacupuncture in poststroke urinary incontinence, resulting in significant variation in efficacy.^[82]^ The absence of comparative studies on different acupoints, manipulation techniques, and treatment durations hampers clinical applicability. It is crucial to propose practical recommendations, establish unified efficacy criteria, and develop standardized clinical protocols to enhance the effectiveness of future electroacupuncture treatments for poststroke urinary incontinence.

If electroacupuncture is deemed acceptable and can reduce the average duration of therapy, it has the potential to reduce healthcare costs and improve patients’ quality of life. However, no economic data was found in the reviewed research.^[83]^ Therefore, future studies should incorporate economic analyses to assess the cost-effectiveness of electroacupuncture in this context.

### 4.5. Limitations

Our study had several limitations. Firstly, we only included Chinese and English literature, excluding studies conducted in well-developed Asian countries such as Japan and Korea. This language limitation prevented us from incorporating relevant research from these countries. Secondly, all the included studies were conducted in China, and the majority of them did not disclose information about funding sources. Consequently, the lack of proper randomization, allocation concealment, and blinding in these studies raises concerns about potential bias in the experimental outcomes. Thirdly, our report lacks information on long-term outcomes and the impact on patients’ quality of life. This is primarily due to the absence of follow-up data in most trials and the limited provision of follow-up information in the included studies.

## 5. Conclusion

Electroacupuncture is a promising treatment for poststroke urinary incontinence, backed by substantial research in China. It has demonstrated effectiveness and safety with no significant adverse effects. Given the range of study designs and claimed treatment effects, however, additional large, well-designed studies and international collaboration are still required for the clinical evaluation of electroacupuncture therapy.

## Author contributions

**Conceptualization:** Yuan-Hao Du.

**Data curation:** Jing Li.

**Methodology:** Yuan-Hao Du, Bo Pang, Bo Li.

**Resources:** Ying Cui, Ji-Peng Yang.

**Software:** Yuping Ma, Junpeng He.

**Writing – original draft:** Tao Jiang, Shiyi Jiang.

**Writing – review & editing:** Yuan-Hao Du, Bo Pang.

## Supplementary Material



## References

[1] Abrams P Andersson KE Birder L; Members of Committees. Fourth International Consultation on Incontinence Recommendations of the International Scientific Committee: Evaluation and treatment of urinary incontinence, pelvic organ prolapse, and fecal incontinence. Neurourol Urodyn. 2010;29:213–40. 20025020 10.1002/nau.20870

[2] Pettersen R Stien R Wyller TB. Post-stroke urinary incontinence with impaired awareness of the need to void: clinical and urodynamic features. BJU Int. 2007;99:1073–7. 17437440 10.1111/j.1464-410X.2007.06754.x

[3] Brittain KR Peet SM Castleden CM. Stroke and incontinence. Stroke. 1998;29:524–8. 9472900 10.1161/01.str.29.2.524

[4] Barrett JA. Bladder and bowel problems after stroke. Rev Clin Gerontol. 2003;12:253–67.

[5] Linsenmeyer TA. Post-CVA voiding dysfunctions: clinical insights and literature review. NeuroRehabilitation. 2012;30:1–7. 22349836 10.3233/NRE-2012-0721

[6] Brittain KR Perry SI Peet SM. Prevalence and impact of urinary symptoms among community-dwelling stroke survivors. Stroke. 2000;31:886–91. 10753993 10.1161/01.str.31.4.886

[7] Mehdi Z Birns J Bhalla A. Post-stroke urinary incontinence. Int J Clin Pract. 2013;67:1128–37. 23834208 10.1111/ijcp.12183

[8] Thomas LH Coupe J Cross LD Tan AL Watkins CL. Interventions for treating urinary incontinence after stroke in adults. Cochrane Database Syst Rev. 2019;2:CD004462. 30706461 10.1002/14651858.CD004462.pub4 PMC6355973

[9] Nambiar AK Bosch R Cruz F. EAU guidelines on assessment and nonsurgical management of urinary incontinence. Eur Urol. 2018;73:596–609. 29398262 10.1016/j.eururo.2017.12.031

[10] Panicker JN. Urogenital symptoms in neurologic patients. Continuum (Minneap Minn). 2017;23:533–52. 28375917 10.1212/CON.0000000000000448

[11] Huang AJ Chesney M Lisha N. A group-based yoga program for urinary incontinence in ambulatory women: feasibility, tolerability, and change in incontinence frequency over 3 months in a single-center randomized trial. Am J Obstet Gynecol. 2019;220:87.e1–87.e13. 10.1016/j.ajog.2018.10.031 PMC6314206 30595143

[12] Rui-zhen T Ya G. Research progress of electroacupuncture in the treatment of urinary incontinence after stroke. J Hebei J Tradit Chin Med. 2014;29:48–51.

[13] Jie-ling L. Overview of urinary incontinence after stroke treated with electroacupuncture in recent years. Inner Mongolia Tradit Chin Med. 2010;29:113–4.

[14] Wei Y. Clinical observation of electroacupuncture Bajiao acupoint combined with tonifying kidney and solidifying acupuncture for the treatment of urinary incontinence after stroke. Clin J Acupunct Moxibustion. 2011;27:37–9.

[15] Fengjun S. Electroacupuncture for post-stroke urinary incontinence:a multi-center randomized controled study. Chin Acupunct Moxibustion. 2013;33:769–73. 24298760

[16] Guangwu W Xuexin S Xiuying T. Observation on the clinical effect of electroacupuncture in the treatment of urinary incontinence after stroke. Clin J Acupunct Moxibustion. 2017;33:22–4.

[17] Munn Z Peters MDJ Stern C Tufanaru C McArthur A Aromataris E. Systematic review or scoping review? Guidance for authors when choosing between a systematic or scoping review approach. BMC Med Res Methodol. 2018;18:143. 30453902 10.1186/s12874-018-0611-x PMC6245623

[18] Arksey H O’Malley L. Scoping studies: towards a methodological framework. Int J Soc Res Methodol. 2005;8:19–32.

[19] Moher D Liberati A Tetzlaff J Altman DG; PRISMA Group. Preferred reporting items for systematic reviews and meta-analyses: the PRISMA statement. PLoS Med. 2009;6:e1000097. 19621072 10.1371/journal.pmed.1000097 PMC2707599

[20] Tricco AC Lillie E Zarin W. PRISMA extension for scoping reviews (PRISMA-ScR): checklist and explanation. Ann Intern Med. 2018;169:467–73. 30178033 10.7326/M18-0850

[21] De Chavez AC Backett-Milburn K Parry O Platt S. Understanding and researching wellbeing: Its usage in different disciplines and potential for health research and health promotion. Health Educ J. 2005;64:70–87.

[22] Richardson WS Wilson MC Nishikawa J Hayward RS. The well-built clinical question: a key to evidence-based decisions. ACP J Club. 1995;123:A12–3. 10.7326/ACPJC-1995-123-3-A12 7582737

[23] Xu-yan Y Wei-bin G. Clinical observation of electroacupuncture treatment of urinary incontinence after stroke. Clin J Acupunct Moxibustion. 2006;22:33–4.

[24] Zhishun L Yi L. Evaluation of the curative effect of electroacupuncture on urinary incontinence after stroke. Shanghai Acupunct Magazine. 2007;26:13–4.

[25] Jian-an Z Tong-sheng S Min L. Yu raised 56 cases of stroke urinary incontinence treated with acupoints. Shaanxi Tradit Chin Med. 2007;28:1383–4.

[26] Feng-jun S Shili Z Yingmin Y. Observation on the clinical effect of electroacupuncture in the treatment of urinary incontinence after stroke. Clin J Acupunct Moxibustion. 2008;24:32–3.

[27] Jingyi C Zhishun L Yi W. Electroacupuncture treatment of 32 cases of acute urinary incontinence after stroke. Global Tradit Chin Med. 2008;1:38–41.

[28] Fengjun S Shili Z Junhui F. Electroacupuncture for 60 cases of urinary incontinence after stroke. Chin Med Sci Technol. 2009;16:417–8.

[29] Jing-tao X Shen-li Z Mei C. Clinical observation of electroacupuncture combined with methylclofenate in the treatment of urinary incontinence after stroke. Inner Mongolia Tradit Chin Med. 2009;28:18–9.

[30] Zhishun L. Observation on the curative effect of electroacupuncture treatment of acute urinary incontinence after stroke. New Tradit Chin Med. 2010;42:73–5.

[31] Qianwen S. Observation on the curative effect of electroacupuncture combined with bladder function training in the treatment of post-stroke urinary incontinence. Chin J Phys Med Rehabil. 2010;32:394–5.

[32] Xiao-dong F Jun-mei B. Clinical observation of electroacupuncture treatment of urinary incontinence after stroke. CJGMCM. 2011;26:321–2.

[33] Bo S Weibin G Lihui H. Observation on the curative effect of electroacupuncture on urinary incontinence after cerebral infarction. Heilongjiang Tradit Chin Med. 2011;40:36–7.

[34] Jia-mei C. Randomized controlled clinical trials for electroacupuncture treatment of urinary incontinence in stroke patients. Acupunct Res. 2011;36: 428–432. 22379789

[35] Xiaoyun C Cong X. Clinical study of electroacupuncture stimulation for the treatment of urinary incontinence after stroke. Hubei J Tradit Chin Med. 2011;33:7–8.

[36] Haiying W Dongxia D Xiaoyuan H. Observation on the effect of electroacupuncture stimulation combined with pelvic floor muscle training in the treatment of 43 cases of urinary incontinence after cerebral infarction. Qilu Nurs Magazine. 2011;17:12–3.

[37] Yaoqi H. Self-made qi Yishen decoction combined with electric acupuncture to treat 43 cases of urinary incontinence after stroke. Chin Med Sci Technol. 2011;18:359–60.

[38] Guoying Z. Observation on the clinical effect of electroacupuncture on 46 patients with urinary incontinence after stroke. Chin J Phys Med Rehabil. 2012;34:462–4.

[39] Ping J. The rehabilitation effect of electric acupuncture combined with acupoint application on urinary incontinence after stroke. Zhejiang J Tradit Chin Med. 2012;47:355.

[40] Qun Z Qiuhong C Bo S. Electroacupuncture combined with pelvic floor muscle exercise to treat 50 cases of stroke urinary incontinence. Chin Med Sci Technol. 2012;19:83–4.

[41] Hui Z. Clinical observation of the foot sensing area of the head acupoint and the treatment of urinary incontinence after stroke with electric acupuncture point. Clin J Acupunct Moxibustion. 2013;29:25–7.

[42] Shandong X Dongmei W. Water acupuncture combined with electric acupuncture point to treat urinary incontinence after stroke. J Cardiovasc Cerebrovasc Dis Tradit Chin Western Med. 2014;12:327–8.

[43] Xiaoyan L Erlin L. Clinical observation of 62 cases of urinary incontinence after stroke treated by Sishnchong and kidney Yuhuiyang electroacupuncture. Chin Disability Med. 2014;22:156–7.

[44] Youyu C. Clinical observation of electroacupuncture treatment of 60 cases of urinary incontinence after stroke. Chin Pract Med. 2015;10:271–2.

[45] Wuyang F Haiyan C. Clinical observation of electroacupuncture combined with salt moxibustion in the treatment of post-stroke urinary incontinence. Clin J Acupunct Moxibustion. 2016;32:8–11.

[46] Jin Z Lingjun H. Clinical observation of electroacupuncture stimulation combined with pelvic floor muscle training in the treatment of patients with urinary incontinence after cerebral infarction. Chin J Tradit Chin Med. 2016;31:3377–80.

[47] Xiao-ya L Jie-yu W Dan W. Clinical observation of high-frequencyelectroacupuncture on post-stroke urinary incontinence. Hebei Tradit Chin Med. 2017;39:1555–8.

[48] Liangjun Z. Clinical observation of electroacupuncture combined with salt moxibustion in the treatment of post-stroke urinary incontinence. Clin Med World. 2017;11:120.

[49] Shengnan Q Ningning S Chunyan Z. Clinical study of electric acupuncture combined with warm acupuncture and moxibustion in the treatment of post-stroke urinary incontinence. Diabetes World. 2019;16:39.

[50] Feng X. Clinical study of electric acupuncture combined with warm acupuncture and moxibustion in the treatment of post-stroke urinary incontinence. Bipods Health Care. 2019;225:31–2.

[51] Yuzhen L. Observation on the clinical effect of box moxibustion combined with electric acupuncture in patients with urinary incontinence after stroke. Chin Foreign Med Res. 2019;17:12–4.

[52] Jiaofeng Y Shufang L Juanjuan Y. Clinical study of electric acupuncture combined with warm acupuncture and moxibustion in the treatment of post-stroke urinary incontinence. Asia Pac Tradit Med. 2019;15:132–5.

[53] Jiajin X Yonglei Z Jie J. Effect of Shenyu Huiyang electroacupuncture combined with pelvic floor muscle training on urinary flow dynamics index and bladder function in patients with post-stroke urinary incontinence. Emerg Dis Tradit Chin Med China. 2019;28:1778–1780,1799.

[54] Qichang D. Observation on the clinical effect of repeated transcranial magnetic stimulation combined with electroacupuncture in the treatment of urinary incontinence after stroke. Electron J Tradit Chin Western Med Combined Cardiovasc Dis. 2020;8:55–6.

[55] Lanqun L. Effects of electroacupuncture at Zhongji and Guanyuan on urge urinary incontinence after stroke. Chin J Rehabil Theory Pract. 2020;26:93–7.

[56] Yuanzheng S. Clinical observation of acupuncture foot sensing area combined with local electric acupuncture for the treatment of urinary incontinence after stroke. Tianjin Tradit Chin Med. 2020;37:71–5.

[57] Feifei L. Urinary kinetic study of electroacupuncture abdominal acupoints for acute urinary incontinence after cerebral infarction. Zhejiang J Tradit Chin Med. 2020;55:240–2.

[58] Chao J Li-Na L. Clinical observation of electroacupuncture combined with moxibustion in the treatment of urinary incontinence after renal yang deficiency stroke. Acupunct Res. 2020;45:578–82. 10.13702/j.1000-0607.200300 32705834

[59] Chen S. Clinical observation of the efficacy of electroacupuncture at“four sacral points”on post-stroke urinary incontinence. J Liaoning Univ TCM. 2020;22:164–8.

[60] Xiangxin Z. The clinical effect of electroacupuncture combined with mango acupuncture in the treatment of post-stroke urinary incontinence and its effect on urinary flow dynamics. J Changchun Univ Tradit Chin Med. 2022;38:757–60.

[61] Xiangxin Z. Observation on the curative effect of electroacupuncture combined with mango acupuncture in the treatment of post-stroke urinary incontinence. Shanghai Acupunct Magazine. 2022;41:889–94.

[62] Xi W. Therapeutic evaluation of electroacupuncture on urge urinary incontinence patients after stroke. Chin J Tradit Chin Med. 2022;37:6180–3.

[63] Guangyu M Guoqing X. Electric acupuncture combined with warm acupuncture to treat 30 cases of urinary incontinence after stroke. Mod Distance Educ Tradit Chin Med China. 2014;12:72–3.

[64] Lanjing L Jingjing G. Clinical study of electric acupuncture combined with warm acupuncture and moxibustion in the treatment of post-stroke urinary incontinence. Dietary Health Care. 2019;6:74–5.

[65] Furong D. Observation on the curative effect of electric acupuncture and warm acupuncture on urinary incontinence after stroke. Heilongjiang Tradit Chin Med. 2020;49:402–3.

[66] Hong-jun W. Electroacupuncture combined with wrist and ankle acupuncture to treat 128 cases of urinary incontinence after stroke. Clin J Acupunct Moxibustion. 2004;20:29–29.

[67] Fengjun S. Electroacupuncture treatment of 218 cases of urinary incontinence after stroke. Chin Med Sci Technol. 2011;18:184.

[68] Li-guo Z. Acupuncture combined with an electric needle to treat 60 cases of urinary incontinence after stroke. Heilongjiang Tradit Chin Med. 2009;38:41–2.

[69] Fengjun S. Electroacupuncture treatment of 218 cases of urinary incontinence after stroke. In: 2011 Zhejiang Provincial Physical Medicine and Rehabilitation Academic Annual Conference and Rehabilitation New Progress Study Class. Ji’an, Zhejiang, China. 2011.

[70] Min Z. Clinical research progress of traditional Chinese and Western medicine on urinary incontinence after stroke. Hebei Tradit Chin Med. 2020;42:1910–5.

[71] Rotar M Blagus R Jeromel M Skrbec M Tršinar B Vodušek DB. Stroke patients who regain urinary continence in the first week after acute first-ever stroke have better prognosis than patients with persistent lower urinary tract dysfunction. Neurourol Urodyn. 2011;30:1315–8. 21488096 10.1002/nau.21013

[72] Eldar R Ring H Tshuwa M Dynia A Ronen R. Quality of care for urinary incontinence in a rehabilitation setting for patients with stroke. Simultaneous monitoring of process and outcome. Int J Qual Health Care. 2001;13:57–61. 11330445 10.1093/intqhc/13.1.57

[73] Ween JE Alexander MP D'Esposito M Roberts M. Incontinence after stroke in a rehabilitation setting: outcome associations and predictive factors. Neurology. 1996;47:659–63. 8797460 10.1212/wnl.47.3.659

[74] Feng QF Zhang A-D Xing M Wang X Ming S-R Chen Y-L. Electroacupuncture alleviates bladder overactivity via inhabiting bladder P2X(3) receptor. Evid Based Complement Alternat Med. 2020;2020:4080891. 32256644 10.1155/2020/4080891 PMC7103056

[75] Min Z. The clinical effect of acupuncture combined with traditional Chinese medicine hot iron in the treatment of urinary incontinence after stroke of renal yang deficiency and its effect on the urodynamics of patients. Clin J Acupunct Moxibustion. 2018;34:25–8.

[76] Zhigao T Wei Z Houwu G. Meta analysis of the clinical effect of electroacupuncture in the treatment of urinary incontinence after stroke. Clin J Acupunct Moxibustion. 2015;31:74–7.

[77] Yang C. Innovative principle and characteristics of acupuncture electroacupuncture instrument. In: The 20th Anniversary of the Establishment of the Acupuncture Equipment Professional Committee of the Chinese Society of Acupuncture and Moxibustion and the 2009 International Symposium on Acupuncture Equipment. Shanghai, China. 2009.

[78] Sjostrom M Umefjord G Stenlund H Carlbring P Andersson G Samuelsson E. Internet-based treatment of stress urinary incontinence: a randomised controlled study with focus on pelvic floor muscle training. BJU Int. 2013;112:362–72. 23350826 10.1111/j.1464-410X.2012.11713.x PMC3798106

[79] Sayre JW Toklu HZ Ye F Mazza J Yale S. Case reports, case series – from clinical practice to evidence-based medicine in graduate medical education. Cureus. 2017;9:e1546. 29018643 10.7759/cureus.1546 PMC5630458

[80] Avery K Donovan J Peters TJ Shaw C Gotoh M Abrams P. ICIQ: a brief and robust measure for evaluating the symptoms and impact of urinary incontinence. Neurourol Urodyn. 2004;23:322–30. 15227649 10.1002/nau.20041

[81] Moher D Hopewell S Schulz KF. CONSORT 2010 explanation and elaboration: updated guidelines for reporting parallel group randomised trials. BMJ. 2010;340:c869. 20332511 10.1136/bmj.c869 PMC2844943

[82] MacPherson H Altman DG Hammerschlag R; STRICTA Revision Group. Revised STandards for Reporting Interventions in Clinical Trials of Acupuncture (STRICTA): extending the CONSORT statement. PLoS Med. 2010;7:e1000261. 20543992 10.1371/journal.pmed.1000261 PMC2882429

[83] Kim HS Choi JW Chang SH Lee KS Oh JY. Treatment duration and cost of work-related low back pain in Korea. J Korean Med Sci. 2005;20:127–31. 15716617 10.3346/jkms.2005.20.1.127 PMC2808558

