# Supplementary material for: Electroacupuncture for poststroke urinary incontinence: A systematic scoping review
Source: Medicine (Baltimore). 2026 Jan 9;105(2):e45884. doi: 10.1097/MD.0000000000045884 (PMC12795040; doi:10.1097/MD.0000000000045884)
Supplement: Supplementary file 1 [file medi-105-e45884-s001.docx]

**S1 File. Preferred Reporting Items for Systematic reviews and Meta-Analyses extension for Scoping Reviews (PRISMA-ScR) Checklist**

| **SECTION** | **ITEM** | **PRISMA-ScR CHECKLIST ITEM** | **REPORTED ON PAGE #** |
| --- | --- | --- | --- |
| **TITLE** | | | |
| Title | 1 | Identify the report as a scoping review. | Page 1 |
| **ABSTRACT** | | | |
| Structured summary | 2 | Provide a structured summary that includes (as applicable): background, objectives, eligibility criteria, sources of evidence, charting methods, results, and conclusions that relate to the review questions and  objectives. | Page 1 |
| **INTRODUCTION** | | | |
| Rationale | 3 | Describe the rationale for the review in the context of what is already known. Explain why the review questions/objectives lend themselves to a scoping  review approach. | Page 2-3 |
| Objectives | 4 | Provide an explicit statement of the questions and objectives being addressed with reference to their key elements (e.g., population or participants, concepts, and context) or other relevant key elements used to  conceptualize the review questions and/or objectives. | Page 3 |
| **METHODS** | | | |
| Protocol and registration | 5 | Indicate whether a review protocol exists; state if and where it can be accessed (e.g., a Web address); and if  available, provide registration information, including the registration number. | None |
| Eligibility criteria | 6 | Specify characteristics of the sources of evidence used as eligibility criteria (e.g., years considered, language, and publication status), and provide a  rationale. | Page 3 |
| Information sources* | 7 | Describe all information sources in the search (e.g., databases with dates of coverage and contact with authors to identify additional sources), as well as the  date the most recent search was executed. | Page 3-4 |
| Search | 8 | Present the full electronic search strategy for at least 1 database, including any limits used, such that it could be repeated. | Page 3-4 |
| Selection of  sources of evidence† | 9 | State the process for selecting sources of evidence  (i.e., screening and eligibility) included in the scoping review. | Page 4 |
| Data charting process‡ | 10 | Describe the methods of charting data from the included sources of evidence (e.g., calibrated forms or forms that have been tested by the team before their use, and whether data charting was done independently or in duplicate) and any processes for  obtaining and confirming data from investigators. | Page 5 |
| Data items | 11 | List and define all variables for which data were sought and any assumptions and simplifications made. | Page 4 |
| Critical appraisal of individual sources of evidence§ | 12 | If done, provide a rationale for conducting a critical appraisal of included sources of evidence; describe the methods used and how this information was used in any data synthesis (if appropriate). | None |
| Synthesis of results | 13 | Describe the methods of handling and summarizing the data that were charted. | Page 4 |
| **SECTION** | **ITEM** | **PRISMA-ScR CHECKLIST ITEM** | **REPORTED ON PAGE #** |
| **RESULTS** | | | |
| Selection of sources of evidence | 14 | Give numbers of sources of evidence screened, assessed for eligibility, and included in the review, with reasons for exclusions at each stage, ideally using a  flow diagram. | Page 5 |
| Characteristics of sources of evidence | 15 | For each source of evidence, present characteristics for which data were charted and provide the citations. | Page 11-13 |
| Critical appraisal within sources of evidence | 16 | If done, present data on critical appraisal of included sources of evidence (see item 12). | None |
| Results of individual sources  of evidence | 17 | For each included source of evidence, present the relevant data that were charted that relate to the  review questions and objectives. | Table 1 |
| Synthesis of results | 18 | Summarize and/or present the charting results as they relate to the review questions and objectives. | Page 11-13 |
| **DISCUSSION** | | | |
| Summary of evidence | 19 | Summarize the main results (including an overview of concepts, themes, and types of evidence available),  link to the review questions and objectives, and consider the relevance to key groups. | Page 13-16 |
| Limitations | 20 | Discuss the limitations of the scoping review process. | Page 16 |
| Conclusions | 21 | Provide a general interpretation of the results with respect to the review questions and objectives, as well as potential implications and/or next steps. | Page 16 |
| **FUNDING** | | | |
| Funding | 22 | Describe sources of funding for the included sources of evidence, as well as sources of funding for the  scoping review. Describe the role of the funders of the scoping review. | Page 17 |

From: Tricco AC, Lillie E, Zarin W, O'Brien KK, Colquhoun H, Levac D, et al. PRISMA Extension for Scoping Reviews (PRISMAScR): Checklist and Explanation. Ann Intern Med. 2018;169:467–473. [doi: 10.7326/M18-0850](http://annals.org/aim/fullarticle/2700389/prisma-extension-scoping-reviews-prisma-scr-checklist-explanation).

**S2 File. The detailed search strategy**

The search strategy for PubMed (7)

((electroacupuncture[MeSH Terms]) OR (electroacupuncture therapy[MeSH Terms]) OR (electric acupuncture)) AND ((stroke[MeSH Terms]) OR (after stroke[MeSH Terms])) AND ((urinary incontinence[MeSH Terms]) OR (involuntary urination[MeSH Terms]))

The search strategy for Web of science (15)

TS=(electroacupuncture* OR electroacupuncture therapy* OR electric acupuncture*)AND TS=(stroke* OR after stroke* OR post-stroke)AND TS=(urinary incontinence* OR involuntary urination* )

The search strategy for Embase (14)

('electroacupuncture'/exp OR 'electroacupuncture therapy'/exp OR ' electric acupuncture '/exp) AND ('stroke'/exp OR 'post-stroke'/exp OR 'after stroke'/exp) AND ('urinary incontinence'/exp OR 'involuntary urination')

The search strategy for Cochrane (21)


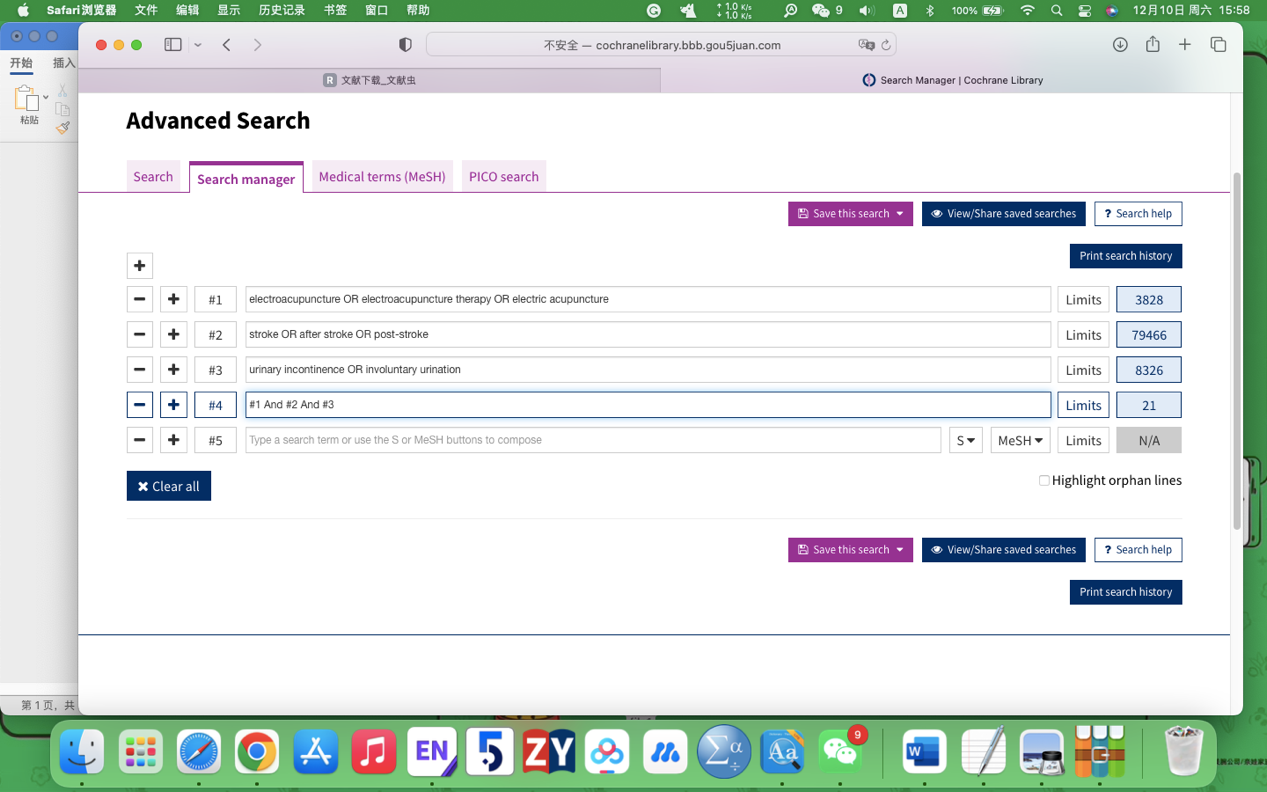


The search strategy for CNKI （123）

(SU='电针' OR SU='电针刺激' OR SU='电针疗法' OR SU='电针治疗') AND (SU='中风' OR SU='卒中' OR SU='脑梗死' OR SU='脑出血') AND (SU='尿失禁' OR SU='小便失禁')

The search strategy for Wanfang （124）

主题:((“电针” or “电针刺激” or “电针疗法” or “电针治疗”) and (“中风” or “卒中” or “脑梗死” or “脑出血”) and ("尿失禁" or “小便失禁”))

The search strategy for VIP （55）

M=(电针 OR 电针刺激 OR 电针疗法 OR 电针治疗 ) AND M=(中风 OR 卒中 OR 脑梗死 OR 脑出血) AND M=(尿失禁 OR 小便失禁)

The search strategy for SinoMed （80）

("电针"[常用字段:智能] OR "电针刺激"[常用字段:智能] OR "电针治疗"[常用字段:智能] OR "电针疗法"[常用字段:智能]) AND ("卒中"[常用字段:智能] OR "中风"[常用字段:智能] OR "脑梗死"[常用字段:智能] OR "脑出血"[常用字段:智能]) AND ("尿失禁"[常用字段:智能] OR "小便失禁"[常用字段:智能])
